# Modified lumbo-pelvic exercise to alleviate mild stress urinary incontinence in middle-aged females

**DOI:** 10.1038/s41598-023-34417-z

**Published:** 2023-05-02

**Authors:** Yi Wang, Liangchao Liu, Qi Chen, Kuiting Gao, Hongchu Wang, Naxin Xu, Yinru Chen, Duo Wai-Chi Wong, Wing-Kai Lam

**Affiliations:** 1 Department of Physical Education, Renmin University of China, Beijing, China; 2 Sports and Social Development Research Center, Renmin University of China, Beijing, China; 3 Physical Education Department, University of International Business and Economics, Beijing, China; 4 School of Physical Education, Shandong University of Technology, Shandong, China; 5 School of Mathematical Sciences, South China Normal University, Guangzhou, China; 6 Sport Science School, Beijing Sport University, Beijing, China; 7 College of Education, Beijing Sport University, Beijing, China; 8 Department of Biomedical Engineering, Faculty of Engineering, The Hong Kong Polytechnic University, Hong Kong, China; 9 Research Institute for Sports Science and Technology, The Hong Kong Polytechnic University, Hong Kong, China; 10 Sports Information and External Affairs Centre, Hong Kong Sports Institute, Hong Kong, China

**Keywords:** Physiology, Health care, Urology

## Abstract

Urinary incontinence is one of the common clinical problems of females passing middle age. Traditional pelvic floor muscle training to alleviate urinary incontinence is too dull and unpleasant. Therefore, we were motivated to purpose a modified lumbo-pelvic exercise training incorporating simplified dancing components with pelvic floor muscle training. The objective of this study was to evaluate the 16-week modified lumbo-pelvic exercise program that incorporated dance and abdominal drawing-in maneuvers. Middle-aged females were randomly assigned into the experimental (*n* = 13) and control (*n* = 11) groups. Compared to the control group, the exercise group significantly reduced body fat, visceral fat index, waistline, waist-hip ratio, perceived incontinence score, frequency of urine leakage, and pad testing index (*p* < 0.05). In addition, there were significant improvements in pelvic floor function, vital capacity, and muscle activity of the right rectus abdominis (*p* < 0.05). This indicated that the modified lumbo-pelvic exercise program can promote benefits of physical training and alleviate urinary incontinence in middle-aged females.

## Introduction

Urinary incontinence (or the loss of bladder control) is a common embarrassing clinical problem in middle-aged females. The incidence of urinary incontinence that is caused by pelvic floor muscle dysfunctions ranged from 30 to 60% among middle-aged females^1^. Therefore, pelvic floor muscle dysfunction is one of the particular concerns among pathogenic factors of urinary incontinence^2^. Existing studies demonstrated a significant association between pelvic floor muscle strength, urinary incontinence, and quality of life^3,4^. Pelvic floor muscle training has been recognized as one of the effective interventions to treat urinary incontinence and improve the quality of life because it is low cost and non-invasive without side effects^3,4^. In addition, the exercise training could promote active pelvic floor muscle contractions for better control urethral pressure to alleviate incontinence conditions^5,6^, which was demonstrated by significant improvements in urinary incontinence scores and quality of life^7,8^. Recent reviews suggested pelvic floor muscle training can reduce urinary leakage episodes, pad usage, and anxiety ^9–11^. Moreover, strengthening pelvic floor muscle can enhance the control capability of the bladder neck and inhibit detrusor contractions to improve urinary incontinence. However, individuals felt dull and had poor contemplation to persist pelvic floor muscle training^8^.

Traditional pelvic floor muscle training is typically performed in prone lying position, which limits the full range motion of hip and thus reduce the training efficiency^12^. Other studies indicated that training in a standing position can induce higher pelvic floor muscle activity level than that in the lying position^13^. With a standing position, the pelvic floor muscle training can be incorporated with dancing components to allow training more enjoyable, engaging, and thus better compliance to attain better training effects^14^. The combination of pelvic tilt and twist in dance-integrated training can stretch and activate the pelvic floor muscles^15–17^, including the groin, perineum, waist, abdomen, chest, and arm muscles^14,18^. Such rhythmic/music training movement can also exploit the coordination of surrounding muscles, including the rectus abdominis, hip adductor, and gluteus maximus muscles^15^. Moreover, the level of muscle training was suggested to be augmented and prolonged by dance movements^18^. Essentially, dance-integrated training was shown to reduce the number and intensity of incontinence, pain and discomfort associated with pelvic floor dysfunction^17,19,20^, and thus improving patient satisfaction^21,22^.

Incorporating rhythmic/music features into movements/training can contribute to better motivational qualities and training outcomes^23^, as previously demonstrated in running^24,25^, rowing^26,27^, cycling ^28^ and walking^29–31^. In addition, exercises with music have been shown to reduce perceived exertion of exercise intensity^32^, improve pleasure^28,32^, regulate breathing rhythm^30^ , and prolong the health benefits of exercise^33^ . Similarly, the pelvic floor muscle training that guided by music was shown to achieve faster rehabilitation than the traditional pelvic floor muscle training without music components^8^. Consequently, dance training with music components would be beneficial to develop the exercise habits for pelvic floor muscles.

However, there were some hurdles in encouraging dance-integrated training^34–36^, since the dance movements are not easy to learn and performed correctly. Furthermore, middle-aged females may have poorer hip flexibility than the younger counterparts^37,38^, resulting in difficulty to comprehend the dancing maneuvers. We proposed the modified lumbo-pelvic exercise incorporated with abdominal drawing-in maneuver (ADIM) to perform the rhythmic hip movement that emphasizes core stability, especially the voluntary contraction and regulation of abdominal and pelvic floor muscles^39,40^. The ADIM training involves isometrically contractions of deep abdominal muscles (transversus abdominis, internal abdominal oblique and external abdominal oblique) that pull the abdominal walls inward to the spine and compress the internal organs upward into diaphragm and downward into pelvic floor ^41,42^. Moreover, the ADIM decreases the excessive lumbar lordosis or anterior tilting of the pelvis through abdominal hollowing ^43^ and simultaneously induces selective contraction of the diaphragm and transversus abdominis to contribute to lumbar stabilization. Furthermore, the synergistic activation of the transversus abdominis muscle and posterior fibers of the internal and external abdominal oblique muscle increases the tension of the thoracolumbar fascia and generates intra-abdominal pressure, which transforms the abdomen into a mechanically rigid cylinder ^44,45^. Therefore, the increase of abdominal pressure would improve lumbar and pelvic stability ^46^. Thoracolumbar fascia connects to the contralateral gluteal and hamstrings muscles via the sacrotuberous ligament ^47^. Concurrent with the coactivation of the contralateral gluteus maximus muscle, the ADIM increases the stability of the lumbo-pelvic-hip complex for higher postural stability ^48^.

Despite the important contribution of core stabilization to urinary functions, the effects of ADIM and/or lumbo-pelvic movement on urinary functions remain unknown. The objective of this study was to purpose a modified lumbo-pelvic rhythmic movement training and investigate its effects on perceived incontinence, urinary functions, and related muscle activities. One of the main features and novelties of the proposed training is the integration of pelvic exercise with dance, which is motivated by the fact that individuals felt bored with traditional pelvic floor muscle training. The significance of this study lies in its potential to improve the acceptance of training, and thus alleviate incontinence conditions and improve the quality of life for middle-aged females. It is hypothesized that the training can reduce perceived incontinence, improve urinary functions, and promote higher muscle activation level for the abdominal and pelvic floor muscles. The findings of this study can provide insights of the rehabilitation protocols for urinary incontinence among middle-aged females.

## Results

### Anthropometric and body composition variables

As shown in Table 1, the two-way ANOVA presented significant interaction effects on body fat (*p* = 0.002), visceral fat index (VFI) (*p* = 0.008), waistline (*p* < 0.001), and waist-hip ratio (*p* = 0.006). Further analysis of the interaction revealed that the experiment group reduced body fat (*p* = 0.001), VFI (*p* = 0.008), waistline (*p* > 0.001), and waist-hip ratio (*p* = 0.002) at 16^th^ week compared with the 0 week, while the control group did not have significant difference before and after training (*p* > 0.05). All participants had significantly lowered the body mass index (BMI) after the exercise program (*p* = 0.017).

**Table 1:** Anthropometric and body composition variables.

	Week	Experimental (N = 13)	Control (N = 11)	*p*-value		
				Time	Group	Interaction
Mass (kg)	0	61.7 ± 9.7	59.5 ± 6.6	0.389	0.678	0.424
	16	60.1 ± 9.4	59.5 ± 6.8			
Body fat (%)	0	20.6 ± 5.9	17.8 ± 5.2	**0.001**	0.272	**0.002**
	16	19.9 ± 5.7	17.8 ± 5.0			
Body mass index (kg/m^2^)	0	23.9 ± 3.1	23.3 ± 2.7	**0.017**	0.745	0.360
	16	23.2 ± 3.0	23.9 ± 3.1			
Visceral fat index	0	90.2 ± 80.2	86.9 ± 83.0	0.102	0.987	**0.008**
	16	84.1 ± 75.5	88.5 ± 83.6			
Waistline (cm)	0	77.9 ± 7.5	81.1 ± 8.2	**0.002**	0.221	**0.000**
	16	76.3 ± 7.6	81.3 ± 8.3			
Hipline (cm)	0	97.6 ± 5.5	95.6 ± 5.7	0.644	0.425	0.323
	16	97.5 ± 4.9	95.8 ± 5.6			
Waist-hip ratio	0	0.80 ± 0.05	0.85 ± 0.06	**0.005**	**0.025**	**0.006**
	16	0.78 ± 0.05	0.85 ± 0.07			

Significant values are in bold.

### Electromyography (EMG) variables

As shown in Table 2, significant interaction was found on the muscle activation level of right rectus abdominis (*p* = 0.001) only. Further analysis indicated that the experimental group reduced the muscle activation level of the right rectus abdominis after the exercise program (*p* = 0.036), while the control group showed a significant opposite effect (*p* = 0.018). The similar effects were not found in the left rectus abdominis. The control group had a significantly higher muscle activation level of left gluteus maximus (*p* = 0.046) and right gracilis (*p* = 0.008). All participants showed a significantly lower activation level of the right gluteus maximus after the exercise program (*p* = 0.027).

**Table 2:** Activation level of pelvic floor muscles (mV) variables.

	Week	Experimental (N = 13)	Control (N = 11)	*p*-value		
				Time	Group	Interaction
Rectus abdominis—left	0	0.13 ± 0.13	0.19 ± 0.18	0.139	0.053	0.139
	16	0.05 ± 0.03	0.19 ± 0.18			
Rectus abdominis—right	0	0.13 ± 0.11	0.09 ± 0.06	0.498	0.413	**0.001**
	16	0.06 ± 0.05	0.14 ± 0.06			
Gluteus maximus—left	0	0.21 ± 0.13	0.24 ± 0.21	0.690	**0.046**	0.308
	16	0.16 ± 0.08	0.26 ± 0.26			
Gluteus maximus—right	0	0.32 ± 0.23	0.23 ± 0.22	**0.027**	0.519	0.170
	16	0.22 ± 0.13	0.20 ± 0.25			
Gracilis—left	0	0.31 ± 0.34	0.42 ± 0.31	0.194	0.204	0.984
	16	0.17 ± 0.07	0.28 ± 0.20			
Gracilis—right	0	0.15 ± 0.15	0.39 ± 0.32	0.371	**0.008**	0.453
	16	0.16 ± 0.11	0.51 ± 0.48			

Significant values are in bold.

### Perceived incontinence, physical function, urination and pulmonary variables

As shown in Table 3, there were significant interactions on the Incontinence Quality of Life Questionnaire (I-QOL) score (*p* = 0.013), International Consultation on Incontinence Questionnaire-urinary incontinence (ICIQ-UI) score (*p* = 0.002), pelvic floor function (*p* = 0.037), leakage urine (*p* = 0.009), pad testing (*p* < 0.001), and vital capacity (*p* < 0.001). Further analysis (Table 4) indicated that the participants in the experimental group significantly reduced the I-QOL (*p* = 0.025), ICIQ-UI (*p* = 0.001) scores, frequency of leakage urine (*p* = 0.008), and pad testing index (*p* = 0.001) after the exercise program, while the control group did not show any differences for all variables (*p* > 0.05). Furthermore, the experimental group improved the pelvic floor muscle function (*p* = 0.001) and vital capacity (*p* < 0.001) after the exercise program, while no significant differences were found in the control group (*p* > 0.05). The main group effect indicated a higher urination frequency in the experimental group than the control group (*p* = 0.025). The main time effect indicated a lower nocturia frequency after the exercise program (*p* = 0.020).

**Table 3:** Perceived incontinence, physical function, urination and pulmonary variables.

	Week	Experimental (N = 13)	Control (N = 11)	*p*-value		
				Time	Group	Interaction
Perceived incontinence						
I-QOL score	0	76.00 ± 12.42	76.00 ± 8.53	0.136	0.176	**0.013**
	16	83.95 ± 8.03	73.86 ± 10.26			
ICIQ-UI score	0	6.08 ± 2.24	5.46 ± 1.56	**0.023**	0.273	**0.002**
	16	3.79 ± 1.75	5.88 ± 1.76			
Physical function						
Bladder functional capacity (ml)	0	390.83 ± 183.31	406.67 ± 99.60	0.513	0.702	0.890
	16	400.67 ± 116.95	421.75 ± 74.36			
Pelvic floor function (uV)	0	77.75 ± 15.62	74.25 ± 17.61	**0.025**	**0.001**	**0.037**
	16	96.43 ± 19.08	69.58 ± 20.09			

Significant values are in bold.

**Table 4:** Urination and pulmonary variables.

Urination variables						
Urination frequency	0	9.16 ± 2.19	7.89 ± 2.04	0.062	**0.025**	0.369
	16	8.05 ± 1.55	8.00 ± 1.70			
Nocturia frequency	0	0.83 ± 0.94	1.08 ± 1.38	**0.020**	0.359	0.501
	16	0.25 ± 0.62	0.75 ± 1.22			
Urinary urgency	0	3.08 ± 4.94	3.17 ± 5.61	0.071	0.441	0.071
	16	0.33 ± 0.65	3.17 ± 6.28			
Leakage urine	0	1.17 ± 1.11	0.92 ± 1.38	**0.009**	**0.021**	**0.009**
	16	0.08 ± 0.29	0.92 ± 1.38			
24 h voided volume (ml)	0	1967.67 ± 438.43	1849.33 ± 797.30	0.873	0.614	0.892
	16	1942.67 ± 410.03	1847.25 ± 467.00			
Single voided volume (ml)	0	213.00 ± 59.98	230.75 ± 45.80	0.122	0.525	0.131
	16	286.50 ± 160.79	229.17 ± 33.34			
Pad testing (g)	0	5.27 ± 3.22	3.67 ± 3.54	**0.000**	0.966	**0.000**
	16	2.16 ± 1.79	3.86 ± 3.51			
Nocturia voided volume (ml)	0	392.83 ± 140.73	444.50 ± 182.63	0.202	0.283	0.328
	16	355.08 ± 102.88	439.33 ± 185.29			
Pulmonary variables						
Vital capacity (ml)	0	2443.00 ± 478.80	2386.00 ± 460.54	**0.000**	0.172	**0.000**
	16	2847.33 ± 412.05	2391.50 ± 449.76			

Significant values are in bold.

## Discussion

Considering the practicality of the dancing movement applied in middle-aged females, we developed a modified lumbo-pelvic coordination exercise, which is a simple rhythmic hip movement that incorporated with ADIM, to encourage core stability, and voluntary contraction of the pelvic floor muscles. This study examined the effects of a 16-week modified lumbo-pelvic coordination exercise training on perceived incontinence, urinary function, and pelvic muscle activation in middle-aged females. Our results indicated that the exercise program can improve anthropometric and body composition; and reduce perceived incontinence, frequency of leakage, and pad testing index. Furthermore, our female participants showed better pelvic floor function and vital capacity after the 16-week exercise program.

Body anthropometric and composition variables are predictors of cardiorespiratory diseases. Our positive findings of these variables aligned with those reported in previous dancing studies in middle-aged or older females^49^. From this perspective, we believed our modified lumbo-pelvic coordination exercise can be comparably effective as other dance exercises. Our proposed exercise can also be easily performed indoor with minimal supervision. The music rhythm helps control the movement speed and balance, allowing participants to control the hip rotation consciously. A previous study found that faster tempo music improved exercise performance through the enhancement of arousal and neural activity^50^, while another study discovered that listening certain tempo of music during physical exercise decreased muscle tension and increased physical strength^50–52^. Further study may consider investigating the influence of music tempo and constant/varying beats on the effects of pelvic floor muscle training and urinary incontinence.

Biomechanically, our modified lumbo-pelvic coordination exercise involved the rhythmic pelvic tilting/rotation in left, right, anterior and posterior directions, which emphasizes core stability, contraction control and regulation of abdominal and pelvic floor muscles^39,40^. The selected muscles were attached along the direction of the iliac crest. Shifting the inertia of the weight (center of mass) could induce a passive hip movement during the exercises. The three muscles (rectus abdominis, gluteus maximus and gracilis) are investigated in this study since they are the major stabilizing muscles for the lumbar spine and pelvic. In fact, there is a close relationship between the musculature of the abdominal and pelvic floor muscles^53^. For instance, activation of the core muscle would generate additional forces to assist the bladder neck muscle, which is a continence mechanism^54^. For patients with urinary inconsistency, there was a significant change in motor unit recruitment in abdominal muscle during trunk stability activities^54^. Moreover, the ADIM can increase intra-abdominal pressure by pulling the abdominal wall through the contraction of transverse abdominal and oblique abdominal muscles^46^, contributing to lumbo-pelvic stabilization^44,55^ and strengthening the synergy of pelvic floor muscles^54^. Our findings showed that our participants demonstrated lower muscle activation of the right rectus abdominis after 16-week training, which suggested that the rectus abdominis muscle became efficient/automatic to the lumbo-pelvic exercises^56^. Strengthening pelvic floor muscles (or better muscle synergy/effectiveness) can also enhance the bladder neck capability and control to prevent urinary incontinence. However, a similar benefit was not observed for the left side. It could be related to the unidirectional exercise procedures (clockwise lumbo-pelvic coordination) across training days^57,58^. The directional effect should be considered in the protocol in the future.

More importantly, our modified lumbo-pelvic movement exercises effectively improved incontinence in middle-aged women, as they reported lower perceived incontinence scores (i.e., I-QOL and ICIQ-UI), lower frequency of leakage urine, and pad testing index and also demonstrated better pelvic floor function and vital capacity. Higher pelvic floor function indicates better conscious control of pelvic floor muscle and bladder neck, which is associated with the increased urethral pressure to reduce urinary leakage^5,6^. Higher perceived incontinence scores indicates an improvement of quality of life and psychosocial health^21,22^. Lower frequency of leakage urine and pad testing index have provided direct evidence to support the fact that the lumbo-pelvic movement exercises effectively resolve the incontinence, similar to other dancing interventions^15,16^. In fact, our training program also improved the pulmonary capability of the participants, which could be an alternative to home-based indoor exercises, especially regarding the social distancing measures during the COVID-19 pandemic.

There are some limitations when interpreting our results. First, only the participants with mild stress urinary incontinence were recruited in this study. The results may not be generalizable to different severities. Second, we did not assess the mental health of the participants in this study. Music and exercises could remedy psychological depression, distress, and anxiety, which might moderate the self-reported outcomes. The assessment of mental health may help further examine the underlying working mechanism including biomechanical, muscle control, perceptual-motor aspects to improve stress urinary incontinence. To date, while most interventions had been proven effective, pragmatic implementation in real-life settings is limited and behavioral research might be necessary to promote future use. In the future, smartphone technology with music, exercise guidance and activity trackers can be integrated into exercise intervention to assess the stages of behavioral change.

In conclusion, a 16-week modified lumbo-pelvic coordination exercise for middle-aged females was introduced and evaluated in this study. The exercise program is simpler than dance training program and could be more engaging than traditional pelvic floor muscle training. Our training program demonstrated improvements in perceived incontinence, urinary function, and pelvic muscle activation.

## Methods

### Participants

We enrolled 30 female participants from convenient samples of the community after screening for eligibility. The inclusion criteria were postmenopausal women aged between 55 and 66 that suffered from mild stress urinary incontinence, characterized by the occurrence of urinary incontinence when coughing or sneezing but did not require daily use of urinary pads^59–61^. All participants shall be physically inactive with an exercise habit less than 20 min a day and not more than three times a week.

The exclusion criteria included gynaecological surgery, any surgery in the abdomen, pelvis or lower extremities in the last ten years, injuries of the lower extremities, pelvis or spine in the past six months, reproductive organs prolapse, cesarean section history, hypertension, cardiac insufficiency and taking estrogen medication. No pelvis or low back pain appeared in the last six months. All participants had obtained the doctor and/or physiotherapist permission prior to participating in the study. Ethical approval was obtained from the research ethics committee of sports science from Beijing Sport University, with reference number 2021054H. All protocols were performed in accordance with the relevant guidelines and regulations of the captioned ethics committee. Informed consent was obtained from all participants.

### Study design

**Figure 1:**
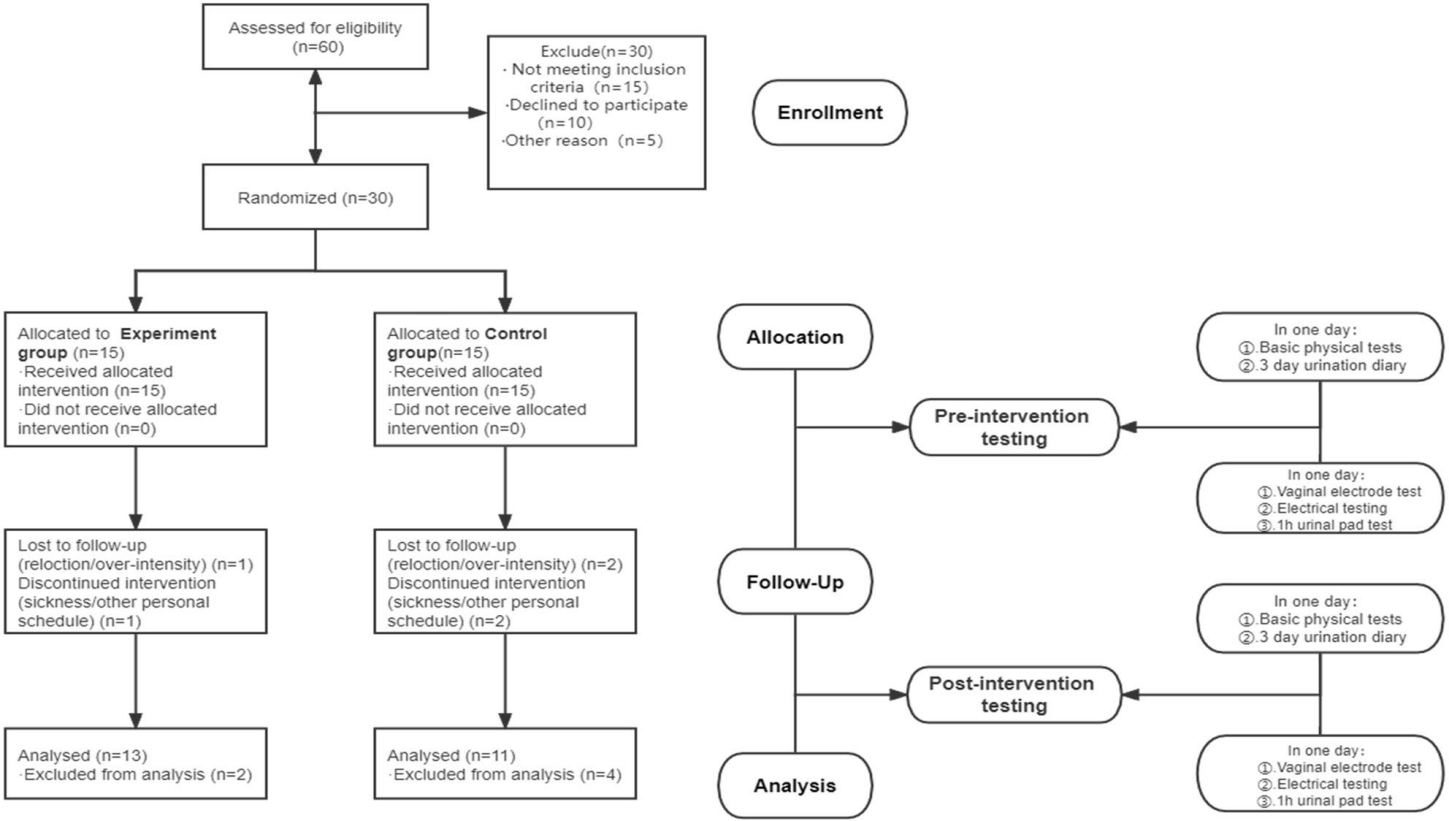
The experimental flowchart.

A total of 60 participants were screened based on the inclusion and exclusion criteria. Fifteen failed to meet the inclusion criteria after further clinical examination, and fifteen refused to participate. Therefore, a total of 30 eligible participants were randomly assigned to the experimental (modified lumbo-pelvic exercise) and control (no exercise) group at an equal allocation ratio. There was no allocation concealment. Six participants lost to follow-up and were excluded from the study. The basic information about the participants is shown in Table 5. There were no significant differences between the experimental and control groups at baseline (*p* > 0.05). The progress of subject enrollment, allocation, intervention, follow-up, and data analysis is illustrated in Fig. 1.

**Table 5:** Basic information between the participants of the experimental and control group.

	Experimental (N = 13)	Control (N = 11)	*P*-value
Mass (kg)	61.7 ± 9.7	59.5 ± 6.6	0.517
Height (cm)	160.9 ± 3.8	161.8 ± 6.7	0.688
Body fat (%)	20.6 ± 5.9	17.8 ± 5.2	0.222
Body mass index (kg/m^2^)	23.9 ± 3.1	23.3 ± 2.7	0.631
Waistline (cm)	77.9 ± 7.5	81.1 ± 8.2	0.330
Hip-line (cm)	97.6 ± 5.5	95.6 ± 5.7	0.397
Physical activity (MET)	2953.8 ± 1159.7	2748.6 ± 1046.9	0.717
Visceral fat index	90.2 ± 80.2	86.9 ± 83.0	0.923

The entire experiment period lasted for 16 weeks. The control group received no exercise interventions and was asked to maintain their usual lifestyle. The experimental group performed the exercise protocol instructed by the same coach.

### Protocol of modified lumbo-pelvic exercise

**Figure 2:**
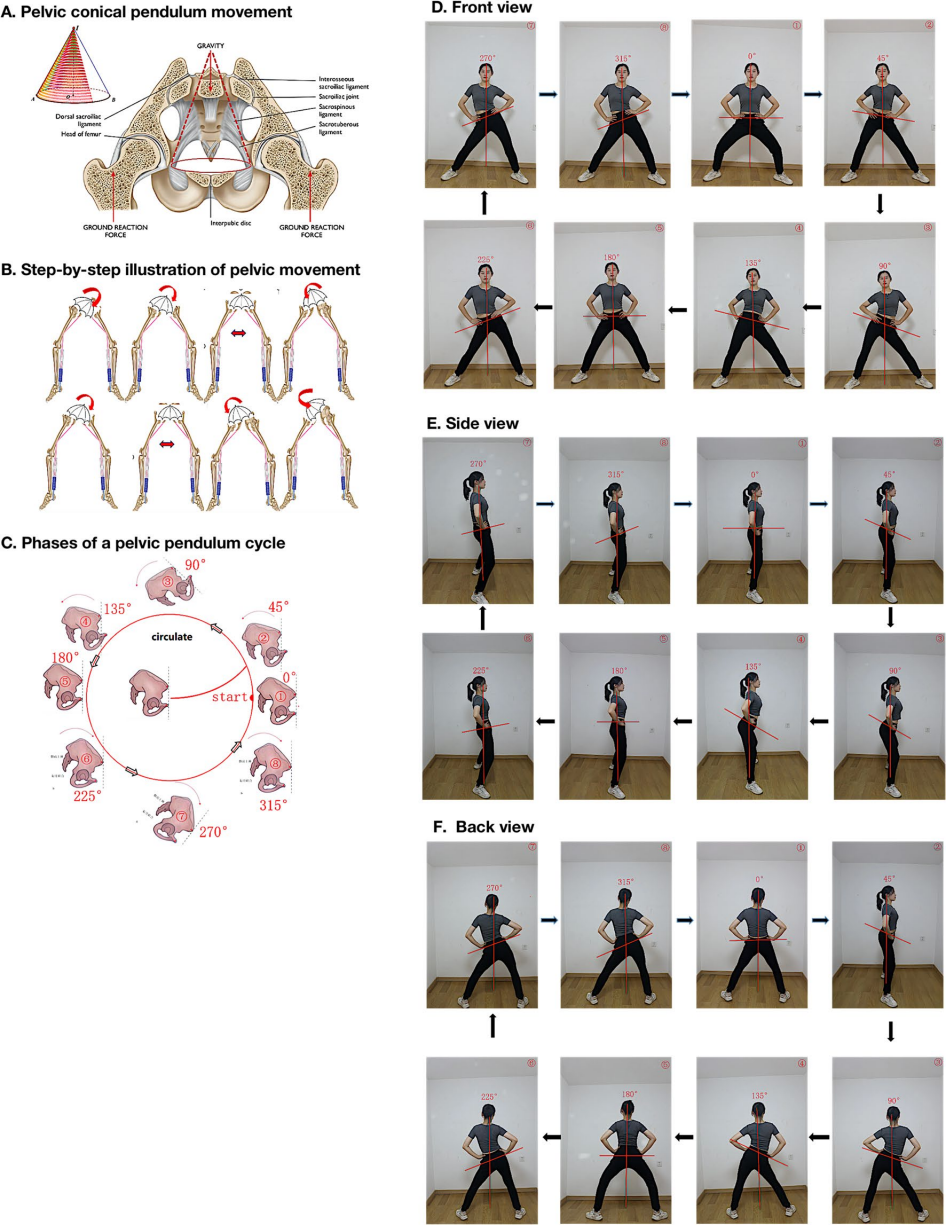
(**A**) Lumbo-pelvic rhythm movement, (**B**) step-by-step illustration of pelvic movement, (**C**) phases of a pelvic pendulum cycle and (**D–F**) lumbo-pelvic rhythm movement in respective views demonstrated by the coach.

Figure 2 illustrates a step-by-step illustration of the exercise moves and explains the lumbo-pelvic movement. Before the exercise, the coach introduced basic breathing techniques to the participants for the exercises. The participants were then instructed to tighten the abdominal/navel to move upward and inward with the pelvic circumduction on the exhale ^21^. They were reminded to consciously control the pelvic floor muscles throughout the hip rotation exercise with following the music beats of the songs.

The participants started with the neutral hip position and then moved in line with the song tempo, in which the beat was voiced out as “2”, “3”, “4”, and “1” by the Mobile Application (Blue Dance App, RuoAn Culture Media company, Shanghai, China). The exercise involved combined pelvic tilt and rotation movement, as shown in Fig. 2. The participants tilted and rotated the pelvic girdle towards the left-anterior, right-anterior, left-anterior, and left-posterior when the beat commanded “2”, “3”, “4”, and “1”. The tilting angle started at 20°, in accordance with the previous study^62^. When the control ability of the pelvic floor muscles improved, the swing and rotating amplitude can be progressively increased.

During each session, the participants performed a 10-min warm-up to focus on joint flexibility in the first two music pieces, followed by 45-min practice with eight music pieces that were randomly presented. The selected songs are available from the smartphone App (Blue Dance App, RuoAn Culture Media, Shanghai, China) that has a built-in popular song list and the corresponding tempo. Only the songs with beats between 56 and 64 bpm were selected in this study, as this tempo matched the preferred body sway and tempo of the participants from our study and previous study ^63^. The hip rotation speed was controlled at the music tempo between 56 and 64 bpm. The playlist of the songs is provided in Table 6.

**Table 6:** Dance song list and musical tempo.

ID	Artist	Song	Type	Year released	Tempo (beat per minute)
1	Westlife	My love	Pop	2000	60
2	Montana Skies	Canyon Breeze	Rumba	2006	64
3	Renliang Qiao	One person one city	Chinese style	2013	64
4	Yiruma	Kiss the rain	Classical	2014	62
5	The Arm	SUMMER	Light music	2004	63
6	Joseph Akins	Little Arwen	Light music	2009	63
7	Oscar Peterson	Georgia on my mind	Jazz	2012	58
8	Kenio Fuke	Espheras	Light music	2011	62

To avoid injury, the participants were asked to rate their perception of nausea after exercises on a 10-point Likert scale (0—asymptomatic; 10—about to vomit; 5 moderately nauseatic) ^64^. In addition, the level of exertion was evaluated using a 10-point sensorimotor Borg Scale after the exercise^65^. The training session was terminated when the nauseatic Likert scale ≥ 5, the Borg scale ≥ 8 or the participants failed to follow the pace. The additional training section would be provided on the following day. The participant would be completely withdrawn from the study if she cannot complete the two training sections consecutively.

### Evaluation

Anthropometric, urination diary, pulmonary capacity, muscle activation, pelvic floor muscle function, and 1-h pad test were conducted before and after the exercise training program. Anthropometric parameters were measured, including body height, weight and body circumferences, body composition, urination diary and pulmonary capacity. Dual-energy X-ray absorptiometry (DXA, Hologic QDR4500W, Inc) was used to measure lean body mass and body fat composition after emptying the stomach, intestines and bladder with minimal radiation^66^. The participants were asked to wear minimally during DXA scans in both supine and prone positions.

For the urination diary, the participants were required to record the time of urination, urine volume, and frequency of urine leakage for three consecutive days. A benchmarked measuring cup (Pyrex, USA) was provided to record the accurate amount of the urine^67^.

For pulmonary capacity, the participants were instructed to stand in a natural and anatomical position, hold the handle of the spirometer (NL-100 spirometer, NaiLi, China), tilt their head slightly back, and breathe in as deep as they could. Then, the participant was asked to breathe out slowly at the mouthpiece until they could not breathe out further. The same procedure was repeated three times, and the largest value was selected for further analyses.

For the muscle activation measurement, Ag/AgCI electrodes with a diameter of 25 mm and inter-electrode distance of 15 mm were placed over the left and right gluteus maximus, rectus abdominis, and gracilis to measure the surface electromyography (EMG) during the lumbo-pelvic coordination exercise. The rectus abdominis electrodes were positioned on the lower part of the muscle, superior to the umbilicus. The gluteus maximus electrodes were placed over the midway between the sacrum and greater trochanter. The gracilis electrodes were positioned on the medial part of the thigh, between the posterior edge of the gracilis, medial muscle of the thigh, and medial edge of the posterior muscles of the lower limb girdle at approximately one-third proximal point. The monopolar reference electrode was positioned on the anterior superior iliac spine for a stable baseline potential. Five consecutive trials were collected for subsequent analyses.

Additionally, pelvic floor muscle function was evaluated using a neuromuscular electrical stimulation device (Phenix USB4, Phenix, France) with eight channels. Vaginal electrodes were applied to assess the pelvic floor muscle functional level. Participants were asked to perform five trials of 3-s maximum pelvic floor muscle contractions in the supine position. The data were bandpass filtered (50 to 1000 Hz), rectified and smoothed^15^.

For the standard 1-h pad test, the participants were asked to wear pre-weighed pads and drink 500 ml of sodium-free liquid in < 15 min^68^. After the preparation of the pad, they were instructed to perform a series of tasks included walking, climbing up and down one flight of stairs, standing up from sitting (10 times), coughing vigorously (10 times), 1-min running on the spot, bending to pick up an object from the floor (5 times), and 1-min washing hands in running water. The weight of the pad was measured with a high-precision balance (Microbalance XP6, Mettler Toledo, Switzerland) before and after the pad test to determine the amount of leakage. The incontinence was classified as mild, moderate, and severe, corresponding to the pad weight increase of 1–10 g, 11–50 g, and > 50 g, respectively ^69^.

### Data and statistical analysis

Outcome measures included anthropometric and body composition variables (body mass, BMI, VFI, waistline, hipline, and waist-hip ratio), EMG (rectus abdominis, gluteus maximus, and gracilis), and assessment of perceived incontinence, physical, urinary, and pulmonary functions.

Data normality of each variable for each group was checked by the Kolmogorov–Smirnov test, while Cook’s distance method was used to check for significant outliers. The Alexander-Govern adjustment was applied if the assumption of homogeneity of variance was violated. Two-way ANOVA mixed design (2 Exercise conditions × 2 Time points) were conducted to examine the main effects of Exercise and Time. The main effect of Exercise or Time was reported only if no significant interaction was determined. The assumption of sphericity was checked by Mauchly’s test and adjusted by Greenhouse–Geisser when it was necessary. All statistical analyses were performed using the R statistical package (R Foundation, Vienna, Austria) with a significance level of 0.05.

## Data Availability

The data that support the findings of this study are available on request from the corresponding author. The data are not publicly available due to privacy of research participants.
